# Evaluation of the Appendicitis Inflammatory Response Score for Patients with Acute Appendicitis

**DOI:** 10.1007/s00268-012-1521-4

**Published:** 2012-03-24

**Authors:** S. M. M. de Castro, Ç. Ünlü, E. Ph. Steller, B. A. van Wagensveld, B. C. Vrouenraets

**Affiliations:** Department of Surgery, St. Lucas Andreas Hospital, Jan Tooropstraat 164, 1061 AE Amsterdam, The Netherlands

## Abstract

**Background:**

Acute appendicitis is still a difficult diagnosis. Scoring systems are designed to aid in the clinical assessment of patients with acute appendicitis. The Alvarado score is the most well known and best performing in validation studies. The purpose of the present study was to externally validate a recently developed appendicitis inflammatory response (AIR) score and compare it to the Alvarado score.

**Methods:**

The present study selected consecutive patients who presented with suspicion of acute appendicitis between 2006 and 2009. Variables necessary to evaluate the scoring systems were registered. The diagnostic performance of the two scores was compared.

**Results:**

The present study included 941 consecutive patients with suspicion of acute appendicitis. There were 410 male patients (44%) and 531 female patients (56%). The area under the receiver operating characteristic curve of the AIR score was 0.96 and significantly better than the area under the curve of 0.82 of the Alvarado score (*p* < 0.05). The AIR score also outperformed the Alvarado score when analyzing the more difficult patients, including women, children, and the elderly.

**Conclusions:**

This study externally validates the AIR Score for patients with acute appendicitis. The scoring system has a high discriminating power and outperforms the Alvarado score.

## Introduction

In 1880, Robert Lawson Tait performed the first appendectomy for appendicitis in England [1]. Now, more than 130 years later, this most common of all surgical diseases can still be a diagnostic problem. This is demonstrated by the high negative laparotomy rates documented in the literature. A study performed in 2005 in the Netherlands found that approximately 15% of the patients underwent a negative appendectomy, a number similar to another large Swedish study [2]. The negative appendectomy rate was 13% in another large North American study [3].

It is safe to assume that the negative laparotomy rate declined to approximately 10% with the routine use of ultrasonography (US) [4]. The higher sensitivity of computed tomography (CT) seems to have had an even greater effect on the negative laparotomy rate, which has decreased even further to 5–10% [4, 5]. In many European countries, most surgeons still consider acute appendicitis to be a clinical diagnosis and do not routinely perform imaging studies [6].

Scoring systems have been designed to aid in the clinical assessment of patients with acute appendicitis. The Alvarado score is the most well known and best performing in validation studies, but it has some drawbacks [7–9]. Its construction was based on a review of patients who had been operated with suspicion of appendicitis, whereas the score is supposed to be used on all patients with suspicion of appendicitis. Also, the score does not incorporate C-reactive protein as a variable, although many studies have shown the importance of C-reactive protein in the assessment of patients with appendicitis [10].

The recently introduced appendicitis inflammatory response (AIR) score was designed to overcome these drawbacks [11]. This score incorporated the C-reactive protein value in its design and was developed and validated on a prospective cohort of patients with suspicion of acute appendicitis.

The objective of the present study was to externally validate the AIR score on a consecutive cohort of patients with suspicion of acute appendicitis and compare the AIR score’s performance to the Alvarado score.

## Methods

The present study selected consecutive emergency room patients with suspicion of acute appendicitis between January 2006 and January 2009. The population consisted of all patients who complained of sudden-onset, non-traumatic abdominal pain. The data of these patients were previously used for a different study evaluating the use of imaging for acute appendicitis [12].

A senior surgical resident initially examined the patients, and the decision to operate was subsequently confirmed by a senior surgical staff member. Imaging by means of US or CT was used selectively in the present study and at discretion of the surgeon. The surgical procedures consisted of either a laparotomy or diagnostic laparoscopy followed by a laparoscopic appendectomy. The diagnosis of acute appendicitis during laparoscopy was established on the basis of macroscopic findings. A macroscopically normal appendix found at laparoscopy was left in situ. The diagnosis of appendicitis was confirmed histologically in all resected specimens. Appendicitis was pathologically diagnosed when infiltration of the muscularis propria by neutrophil granulocytes was seen [13]. Patients were classified into two groups: (1) phlegmonous appendicitis and (2) advanced appendicitis, defined as a macroscopic gangrenous appendix with or without perforation. A periappendicular abscess confirmed on CT was defined as an appendix that is surrounded by a fluid collection and extensive tissue infiltration, which prevents spread of infection into the free abdominal cavity.

Variables recorded to evaluate the scoring systems include nausea, vomiting, anorexia, migration of pain to the right lower quadrant (RLQ), pain in the RLQ, rebound tenderness, muscular defense, body temperature, high white blood cell (WBC) count, proportion of polymorphonuclear leukocytes, and a high level of C-reactive protein (CRP). These variables are necessary to calculate both the Alvarado score and the AIR score. The two scores are based on different variables, with different points assigned to each variable. In the pediatric population, the child’s history obtained from the parents was used if the patient was too young to give a complete history. An overview of the scoring system is given in Table 1.

**Table 1:** Characteristics of the appendicitis inflammatory response (AIR) score and the Alvarado score^a^

Diagnosis	Alvarado score	AIR score
Vomiting		1
Nausea or vomiting	1	
Anorexia	1	
Pain in RLQ	2	1
Migration of pain to the RLQ	1	
Rebound tenderness or muscular defense	1	
Light		1
Medium		2
Strong		3
Body temperature >37.5°C	1	
Body temperature >38.5°C		1
Leukocytosis shift	1	
Polymorphonuclear leukocytes		
70–84%		1
≥85%		2
WBC count		
>10.0 × 10^9^/l	2	
10.0–14.9 × 10^9^/l		1
≥15.0 × 10^9^/l		2
CRP concentration		
10–49 g/l		1
≥50 g/l		2
Total score	10	12

^a^Alvarado score: sum 0–4 = not likely appendicitis, sum 5–6 = equivocal, sum 7–8 = probably appendicitis, sum 9–10 = highly likely appendicitis. Acute appendicitis response score (AIR): sum 0–4 = low probability, sum 5–8 = indeterminate group, sum 9–12 = high probability

*RLQ* right lower quadrant, *CRP* C-reactive protein, *WBC* white blood cell

Statistical analysis was performed with SPSS statistical software (SPSS Inc, Chicago, IL). A *p* value of <0.05 was considered statistically significant. Pearson’s chi-square test was used to test if differences between dichotomous groups were significant. Fisher’s exact test was used when a table had a cell with an expected frequency of less than 5. The area under the receiver operating characteristic (ROC) curves was used to examine the performance characteristics of the two scoring systems.

## Results

The present study included 941 consecutive patients with suspicion of acute appendicitis. There were 410 male patients (44%) and 531 female patients (56%). General patient characteristics are shown in Table 2. The present cohort was older compared to the original AIR cohort. Otherwise, the two cohorts compared remarkably well. The mean patient age was 32 years, with a range of 1–97 years. Of the 941 patients, 201 (21%) were younger than 18 years of age.

**Table 2:** Patient characteristics

	AIR cohort	Present cohort
	(*n* = 545)	(*n* = 941)
Male/female	250 (46%)/295 (54%)	410 (44%)/531 (56%)
Mean age, years	25	32
No. of patients with appendicitis	191 (35%)	346 (37%)
Phlegmonous	117 (22%)	244 (26%)
Advanced	74 (14%)	102 (11%)
No. of patients who underwent surgery	250 (46%)	435 (46%)
Negative appendectomy (at surgery)	59/250 (24%)	89^a^/426 (21%)

^a^Negative appendectomy consists of patients with no appendicitis at pathological examination (*n* = 41), patients with an alternate diagnosis (*n* = 48)

Overall, 346 of the 941 patients (37%) had appendicitis: 244 patients had pathologically proven phlegmonous appendicitis, and 92 had pathologically proven advanced appendicitis. Another 10 patients had a periappendicular abscess. These 10 patients were classified as part of the advanced appendicitis group, resulting in a total of 102 patients with advanced appendicitis. Of the remaining 595 patients (63%) with no appendicitis, an alternate diagnosis was found in 220 patients (Table 3). At operation, a pathologically normal appendix without an alternate diagnosis was found in 41 patients (4%). Nonspecific abdominal pain was found in the remaining 334 patients. All patients underwent routine follow-up and did not receive antibiotics unless an alternate diagnosis indicated antibiotic use.

**Table 3:** Patients with an alternate diagnosis

Diagnosis	After follow-up	At surgery
	(*n* = 172)	(*n* = 48)
Urinary tract infection	37	
Gastroenteritis	25	
Pelvic inflammatory disease/abscess	17	31
Constipation	13	
Ulcerative colitis/Crohn’s disease	9	8
Diverticulitis	9	5
Gallbladder disease	8	
Ovarian torsion	7	
Upper urinary tract infection	7	
Pancreatitis	5	
Urolithiasis	4	
Ileus	3	
Ovulation bleeding	3	
Lymphadenitis mesenterica	2	
Colon tumor	2	
Other^a^	21	4

^a^Including dysmenorrhea, endometrial cyst, hepatocellulcar carcinoma, strangulated inguinal hernia, bowel ischemia, leiomyoma, multiple myeloma, necrotic uterus myoma, neurinoma, ovarian tumor, perianal abscess, corpus luteum cyst, pneumonia, prostate cancer, prostatitis, pseudomembranous colitis, urachal cyst, Meckel’s diverticulum, familial Mediterranean fever, omental infarction, and two patients with ectopic pregnancy

The area under the ROC curve of the AIR score was 0.96 and significantly better than the area under the curve of 0.82 of the Alvarado score (*p* < 0.05). The AIR score also outperformed the Alvarado score in the analysis of the more difficult to diagnose patients, including women, children, and the elderly (Table 4).

**Table 4:** Discriminating capacity of the AIR score compared to the Alvarado score, according to patient gender and age using receiver operator characteristic (ROC) curve analysis

	No. of patients	AIR score	Alvarado score	*p* Value
Overall	941	0.96	0.82	<0.001
Advanced appendicitis^a^	717	0.96	0.82	<0.001
Gender				
Men	410	0.95	0.79	<0.001
Women	531	0.96	0.82	<0.001
Age, years				
<18	201	0.96	0.80	<0.001
18–49	600	0.97	0.88	<0.001
≥50	140	0.92	0.75	<0.001

^a^Excluding 224 patients with phlegmonous appendicitis

A score of greater than 4 points gave a similar sensitivity for the AIR score and the Alvarado score (0.93 vs. 0.90, respectively) but gave a much higher specificity (0.85 vs 0.55, respectively) (Table 5). This corresponds to a negative predictive value of 0.95 for the AIR score compared to 0.90 for the Alvarado score. Five hundred thirty-three of the 941 patients (57%) were classified by the AIR score to the low-risk group with fewer than 5 scoring points, including 18 patients with phlegmonous appendicitis and 7 with advanced appendicitis (Table 6). The corresponding result for the Alvarado score was 359 patients (38%), including 27 phlegmonous appendicitis patients and 8 advanced appendicitis patients. Of the 595 nonappendicitis patients, the AIR score correctly classified 508 patients (85%) to the low-risk group, compared to 324 patients (55%) for the Alvarado score.

**Table 5:** Diagnostic characteristics of the AIR score and Alvarado score according to the cutoff points

	AIR score		Alvarado score	
Diagnostic value	>4 points	>8 points	>4 points	>8 points
All appendicitis				
Sensitivity	0.93	0.10	0.90	0.29
Specificity	0.85	1.00	0.55	0.95
PV+	0.79	1.00	0.53	0.77
PV–	0.95	0.66	0.90	0.70
Advanced appendicitis				
Sensitivity	0.93	0.24	0.92	0.35
Specificity	0.85	1.00	0.55	0.95
PV+	0.52	1.00	0.26	0.55
PV–	0.99	0.88	0.98	0.90

*PV+* positive predictive value, *PV−* negative predictive value

**Table 6:** Distribution according to the diagnostic test zone and diagnosis for the AIR score and the Alvarado score

Diagnostic test zone	AIR score	Alvarado score
Score >8	36	130
Advanced appendicitis	24	36
Phlegmonous appendicitis	12	64
Negative appendectomy	0	21
Nonoperated	0	9
Score 5–8	372	452
Advanced appendicitis	71	58
Phlegmonous appendicitis	214	153
Negative appendectomy	48	58
Nonoperated	39	183
Score <5	533	359
Advanced appendicitis	7	8
Phlegmonous appendicitis	18	27
Negative appendectomy	41	10
Nonoperated	467	314

A score greater than 8 points had a lower sensitivity for appendicitis for the AIR score compared with the Alvarado score (0.10 vs. 0.29). However, this was associated with a higher specificity (1.00 vs. 0.95, respectively). These scores translate to a positive predictive value of 0.77 and 1.00 for the AIR and the Alvarado scores, respectively. The AIR classified 36 patients to the high-risk group. All of them had appendicitis. The corresponding figure for the Alvarado score was 130 patients, 100 of whom had appendicitis.

The AIR score classified 41 of the 89 negative appendectomies (46%) to the low probability group and none to the high probability group, compared to 10 patients (11%) and 21 patients (16%), respectively, for the Alvarado score.

If the AIR score had hypothetically been implemented in evaluating the present cohort, the data would translate into 533 patients (57%) who would have been observed as outpatients and spared further diagnostic work-up. Twenty-five of these patients (5%) (18 patients [3%] with phlegmonous appendicitis and 7 patients [1%] with advanced appendicitis) would have been missed but probably discovered during routine follow-up the next day, as their score got higher. Thirty-six patients with a high probability of appendicitis (4%) could undergo direct surgery without any negative appendectomies. The remaining 372 patients (40%) would fall in the intermediate group and would undergo diagnostic imaging, thus safely preventing costly imaging in 544 (941−(372+25)) patients (58%).

## Discussion

The present study shows that the AIR score has a good statistical discrimination for patients with acute appendicitis and outperforms the Alvarado score. The discriminatory property of the AIR score remains high in the more difficult to diagnose patients (e.g., women, children, and the elderly).

Nowadays, the use of US or CT in patients suspected of having appendicitis is common. However, imaging does not perform well in patients with low and high prevalence of the disease, and CT should be used selectively to minimize exposure to ionizing radiation [14]. Moreover, false negative results may delay surgery and subsequently increase morbidity [15].

A clinical scoring system estimates the probability of appendicitis in a patient and should aid in the decision-making process for treatment because of its simple design and application. There are a number of reasons to use scoring systems in managing cases of appendicitis. A clinical score may be suitable as an instrument for selecting patients for immediate surgery, further examination with imaging techniques, or observation. The score can be repeated during active observation and influence the decision to operate. It must be emphasized that the intent of the scoring system is not to establish a primary diagnosis of appendicitis, but simply to discriminate objectively when there is uncertainty.

Another reason to use such a scoring system is to better describe the patients that are included in clinical studies and thereby facilitate the comparison of results. Many studies performed in patients with appendicitis suffer from selection bias. For instance, two recent studies that compared the use of antibiotics to routine surgery for patients with acute appendicitis reported favorable results with the antibiotic treatment [16, 17]. Unfortunately, the severity of disease in these patients was unclear because the decision to cross over to the surgery group was left to the surgeon. The possible bias in severity of disease was the main critique of these studies after their publication [18–22]. Similarly, the value of diagnostic laparoscopy is dependent on many factors, but it also is limited to the types of patients enrolled in a particular study and thus the prevalence of the disease in a particular population. For instance, including many suspicious cases would lower the yield of laparoscopy significantly, whereas randomly selecting patients with abdominal pain would increase the yield significantly. A validated scoring system can aid in better comparing the results of these studies.

A study on malpractice lawsuits from North America found that appendicitis ranks third among lawsuits, even though appendicitis is the cause of acute abdominal pain only about 5% of the time or less [23]. An objective validated scoring system could legally strengthen decisions made in the emergency room and could avoid malpractice liability. Most claims involve misdiagnosis or delayed diagnosis, and common pitfalls include poor documentation.

The most commonly known scoring system is the Alvarado score. The Alvarado score was first reported in 1986 and was based on the weight of several significant variables found in 305 patients with acute appendicitis. Other variations on the Alvarado score have also been developed but do not differ much [24, 25]. These scoring systems never enjoyed wide application because of their suboptimal discriminatory properties. The AIR score was first reported in 2008. It was based on data collected prospectively from 545 patients admitted for suspected appendicitis at four hospitals. The score was developed on 316 randomly selected patients and evaluated on the remaining 229 patients. It was based on similar values to the Alvarado score, but it also included C-reactive protein as a new variable. A recent meta-analysis showed that when both an elevated WBC count and elevated C-reactive protein level are present, there is a fivefold increase in the positive likelihood ratio for acute appendicitis [10].

Routine use of an Alvarado-like scoring system was evaluated in a large German study comparing 870 patients who did not receive routine scoring with 614 patients who were evaluated with a Alvarado-like scoring system [26]. The scoring system consisted of eight variables developed in another study and validated on a Dutch population [24]. The scoring system also did not include C-reactive protein, and it found no difference in the rates of perforated appendix, negative appendectomy, or complications between groups. However, it did find a significantly lower delayed appendectomy rate (2 vs. 8%) and a lower delayed discharge rate (11 vs. 22%) in the group that routinely used the scoring system.

A conditional strategy with CT only after negative or inconclusive US yielded a sensitivity of 94% in a recent study of patients with acute abdominal pain [27]. In the present cohort, 372 patients (40%) would fall in the intermediate group, and, hypothetically, if they all underwent imaging with this strategy, there would be 22 patients (2%) with a negative appendectomy. Thus the negative appendectomy rate could potentially decline from 10% in the present cohort to 2% in the present cohort with the AIR scoring system.

The AIR score probably works better in the pediatric population than the Alvarado score because the variables scored are easy to apply to children. The Alvarado score requires children to identify nausea, anorexia, and migration of pain. This is probably the reason why the Alvarado score compares best to the AIR score in the adolescent age group, because this group closely mimics the initial cohort on which the Alvarado score was designed.

The management of patients with suspected acute appendicitis is still challenging, and the optimal management strategy is still unknown, even after the introduction of US, CT, and diagnostic laparoscopy. This study externally validates that the AIR score has a high discriminating power and outperforms the Alvarado score. This score could aid in selecting patients who require timely surgery or those who require further evaluation. Finally, the score could safely avoid hospitalization and unneeded investigations in patients in whom the diagnosis is unlikely. Such a scoring system is important for future research to better compare results. But first, a proper prospective randomized controlled trial evaluating the effect of introducing such a score in a relevant patient population has to be performed.
